# Supplementary material for: Complexome profiling on the Chlamydomonas lpa2 mutant reveals insights into PSII biogenesis and new PSII associated proteins
Source: J Exp Bot. 2021 Aug 26;73(1):245–62. doi: 10.1093/jxb/erab390 (PMC8730698; doi:10.1093/jxb/erab390)
Supplement: erab390_suppl_Supplementary_Dataset_S1 [file erab390_suppl_supplementary_dataset_s1.zip › Supplemental Dataset 1 - Excel List and all profiles/plots/GAP3_Cre01.g010900.html]

Trivial name: GAP3  
  
Euclidean distance: 130248.98  
Mean Intensity (WT): 8815.31  
Mean Intensity (Mut): 13905.02  
Distance: 9.37  
  
MapMan: glycolysis.cytosolic branch.glyceraldehyde 3-phosphate dehydrogenase (GAP-DH);PS.calvin cycle.GAP  
  
p value of intensity sums Welch test: 0.2637
